# Mediastinal abscess, an unusual way of presentation of eosinophilic esophagitis

**DOI:** 10.1186/s13223-018-0313-2

**Published:** 2019-02-28

**Authors:** Paul Gisasola, Ainara Iriarte, Martha Rosa Larez, Laura Casanova, Luis Bujanda

**Affiliations:** 1 Zumarraga Ospitalea, OSI Goierri-Urola Garaia, Department of Gastroenterology, Hospital Zumarraga, Argixao Auzoa, 20700 Zumarraga, Gipuzkoa, Basque Country Spain; 2 Department of Gastroenterology, Instituto Biodonostia, Centro de Investigación Biomédica en Red en Enfermedades Hepáticas Y Digestivas (CIBERehd), Universidad del País Vasco (UPV/EHU), San Sebastián, Spain

**Keywords:** Eosinophilic esophagitis, Esophagitis, Eosinophilic, Mediastinal abscess

## Abstract

Eosinophilic esophagitis (EoE) is one of the most prevalent esophageal diseases and the leading cause of dysphagia and food impaction in children and young adults. EoE represents a chronic, local immune-mediated esophageal disease, characterized clinically by symptoms related to esophageal dysfunction and histologically by eosinophil-predominant inflammation. Mediastinal abscess is an uncommon condition that typically appears after esophageal perforations or thoracic surgeries, usually requiring treatment for surgical intervention due to its high morbidity–mortality. Mediastinal abscess, outside these two contexts, is extremely rare. We present the case of a mediastinal abscess secondary to EoE. It is important to think about this entity when there is a mediastinal abscess without trauma or previous surgery.

## Introduction

Eosinophilic esophagitis (EoE) is one of the most prevalent esophageal diseases and the leading cause of dysphagia and food impaction in children and young adults. EoE represents a chronic, local immune/antigen-mediated esophageal disease, characterized clinically by symptoms related to esophageal dysfunction and histologically by eosinophil-predominant inflammation [1, 2].

The incidence of EoE has increased in recent years. The infiltration of eosinophils can affect any part of the digestive tract [3, 4].

We present a patient with recurrent mediastinal abscess due to EoE.

## Case report

We present the case of a 26-year-old male who is referred to the digestive consultation by two episodes of spontaneous paraesophageal abscess in an interval of 2 years.

**Fig. 1:**
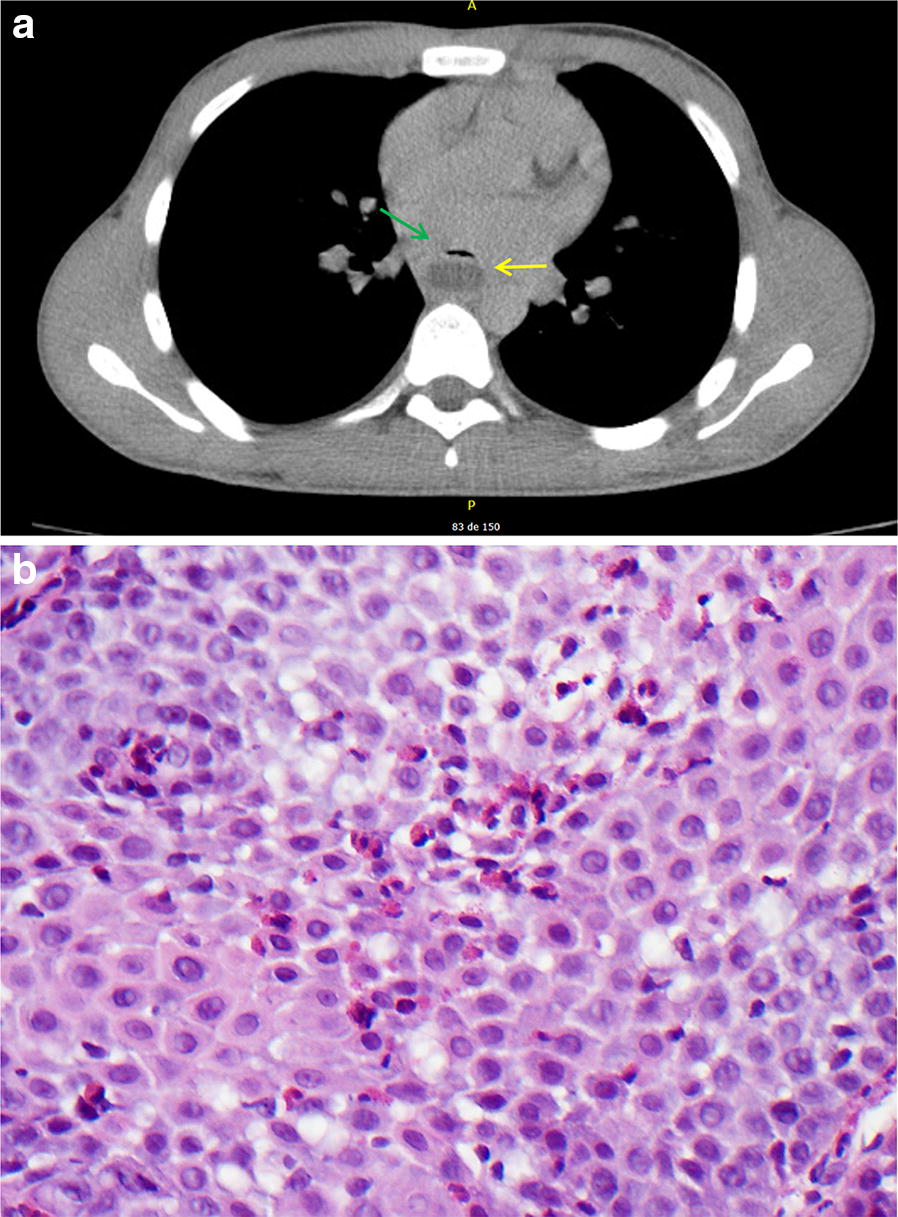
**a** CT image with the mediastinal abscess (yellow arrow) behind the esophagus (green arrow). **b** Pathological findings showing diffusely infiltrated eosinophils, with microabscess formation

It is a patient with no pathological history of interest that is presented in the Emergency Service for dysphagia for solids of 3 days of evolution that at the same time was suffering stabbing chest pain and fever of up to 38.8 °C in the last 24 h. In the last year the patient had already been in the Emergency Room (ER) twice for chest pain with non-altered complementary tests. The patient denies having any traumatic history or onset of symptomatology after food impaction. The physical examination shows no abnormality on a hemodynamically stable patient. It is performed a blood test showed a C reactive protein (CRP) 190 mg/L (Normal values 0–5 mg/L), and white blood cells 12,000/μL (Normal values 4000–10,000). For that reason it is decided to perform thoracic-abdominal computed tomography (CT), where a collection of 8 × 4 × 5 cm is displayed in the third inferior–posterior of the esophagus compatible with hematoma vs mediastinal abscess (Fig. 1).

The surgery service is contacted and it is decided to choose the conservative treatment with broad-spectrum antibiotics and absolute diet. During the admission, a echocardiogram with normal results was performed, an esophagogram that does not present alterations and a gastroscopy, where a linear ulcer of 5 mm in distal third of esophagus with biopsy that shows granulation tissue was found.

The patient is discharged 7 days after, with the normalization of his analytical and clinical parameters, and showing a correct oral tolerance for later control in consultations.

An outpatient USE is requested 3 weeks later, after being discharged, where no paraesophageal collection is displayed. Gastroscopy was repeated where the esophageal ulcer is not visualized and biopsies are taken from the distal and proximal esophagus. In those biopsies, it is noticed an eosinophilic inflammatory infiltration of 40 eosinophils per field.

The patient does not attend any control, so no treatment is started.

One year later the patient returns to the emergency department with chest pain and dysphagia with same characteristics, and elevation of CRP and white blood cells. Again, a toraco-abdominal CT is performed, objectivizing mediastinal collection in the same location as 1 year before, with a size of 7 × 4 × 4 cm, compatible with abscess, which is retreated in a conservative manner with broad spectrum antibiotics. After 10 days, a CT control confirms resolution of the collection.

Ambulatory gastroscopy is performed with biopsy-taking by objectivizing an eosinophilic inflammatory infiltrate compatible with eosinophilic esophagitis.

The patient denies dysphagia, chest pain, heartburn or any other clinic between episodes of mediastinal abscess.

It starts treatment with proton pump inhibitor in double doses during 8 weeks, persisting the eosinophilic inflammatory infiltrate in the biopsies. It is agreed a diet with the patient where two foods will be removed (milk and wheat), obtaining histological remission, and identifying the milk as the cause of the inflammation.

After 2 years of follow-up, the patient maintains milk and derivatives restriction, and has not shown again any episodes of mediastinal abscess.

## Discussion

Mediastinal abscess is an uncommon condition that typically appears after esophageal perforations or thoracic surgeries, usually requiring treatment for surgical intervention due to its high morbidity–mortality [5]. Mediastinal abscess, outside these two contexts, is extremely rare.

The diagnosis is based on a clinical picture compatible with chest pain, fever, and hemodynamic instability according to the general affectation and a compatible technique of image.

The EoE as the cause of this disease is exceptionally rare.

The incidence of EoE has been estimated at 1–20 cases/10,000/year with an estimated prevalence in 13–49 cases/100000 inhabitants/year. This is the second cause of esophagitis in young patients, and is more prevalent in males [1, 2].

For its diagnosis, requires a compatible clinical picture and inflammatory infiltrate Eosinophilic > 15 eosinophils/field. Hematoxylin–eosin (HE) staining is sufficient for histological assessment of EoE in routine clinical practice.

In young and adult patients it is usually presented as a dysphagia for solids, with food impaction and chest pain, while in younger patients it usually has a reflux clinic, vomiting and abdominal pain [1, 2].

The natural evolution of the EoE involves the persistence of symptoms, with the appearance of stenosis, functional anomalies and important impact on the quality of life related to the health of the patient, so the recognition of this entity and its treatment is essential.

Although PPI therapy is useful in a large number of patients to induce remission and maintenance, it was not effective for our patient. For that reason, we decided to go with the diet where were removed two foods, which has achieved a histological remission with the disappearance of the symptomatology.

In summary, we present the case of a mediastinal abscess secondary to EoE. It is important to think about this entity when there is a mediastinal abscess without trauma or previous surgery.
